# How should we treat uncomplicated subacute type B aortic dissection in octogenarians?

**DOI:** 10.1186/s13019-019-0869-z

**Published:** 2019-02-26

**Authors:** Ken Nakamura, Tetsuro Uchida, Azumi Hamasaki, Mitsuaki Sadahiro

**Affiliations:** 1 Second Department of Surgery, Yamagata University Faculty of Medicine, Yamagata, Japan; 2 Division of Cardiovascular Surgery Nihonkai General Hospital, 30 Akihochou, Sakata city, Yamagata, Japan

**Keywords:** Uncomplicated subacute type B aortic dissection, Conservative treatment, Octogenarian, TEVAR, Chronic aortic dissection

## Abstract

**Background:**

Preemptive thoracic endovascular aortic repair (TEVAR) is an advanced treatment that has possibility to improve late outcomes in patients with subacute type B aortic dissection. However, it may not be the treatment of choice for elderly patients with uncomplicated subacute type B aortic dissection because of their inherent frailty and increased risk of periprocedural complications.

**Methods:**

Data were collected between July 2004 and October 2017 in Yamagata university hospital and between February 2016 and May 2018 in Nihonkai General hospital. A total of 152 medically treated subacute type B aortic dissection patients were enrolled in this study. Patients were divided into two groups: age 80 year and older group (Group O, *n* = 33, 22%) and a group < 80 years of age (Group U, *n* = 119, 78%).

**Results:**

During follow-up, the incidence of adverse events was 27% (*n* = 9) in Group O and 37% (*n* = 44) in Group U (*P* = 0.409). The incidence of aortic rupture was 3% (*n* = 1), and the incidence of acute type A dissection was 3% (*n* = 1) in Group O. In Group O, only one patient (3%) died of aorto-bronchial fistula. The Group O patients had less surgical intervention (3 patients [9%] in Group O and 30 patients [25%] in Group U, *P* = 0.047), but aortic related death did not differ between the two groups. The 1-, 2-, and 5-year freedom from aorta-related death rates of Group O were 97, 97, and 97%, respectively, compared with 99, 94, and 91%, respectively, in Group U (*P* = 0.880).

**Conclusions:**

Patients aged 80 years and older who underwent medical treatment for acute and subacute type B dissection had excellent outcomes in chronic phase. The elderly patients had less surgical intervention, but aortic related death did not differ from younger patients.

## Background

Patients with uncomplicated acute type B aortic dissection are typically managed medically with antihypertensive therapy. However, these patients remain at risk for aortic-related complications in the long term, such as aneurysmal degeneration or rupture. Preemptive thoracic endovascular aortic repair (TEVAR) is an advanced treatment that has possibility to improve late outcomes in patients with subacute type B aortic dissection [1, 2]. However, it is unclear whether these improved results in elderly patients with subacute type B aortic dissection translate into improved long-term survival. Optimal medical therapy has produced acceptable late outcomes [3], and increasing age is associated with a decreased occurrence of aortic-related events [4, 5]. Preemptive TEVAR may not be the treatment of choice in elderly patients with uncomplicated type B aortic dissection because of their inherent frailty and increased risk of periprocedural complications. This study examines the short-term and long-term outcomes of octogenarians treated with optimal medical therapy for uncomplicated acute type B aortic dissection.

## Materials and methods

Approval from the Yamagata University Hospital Ethical Committee and patient written consent were obtained.

**Fig. 1:**
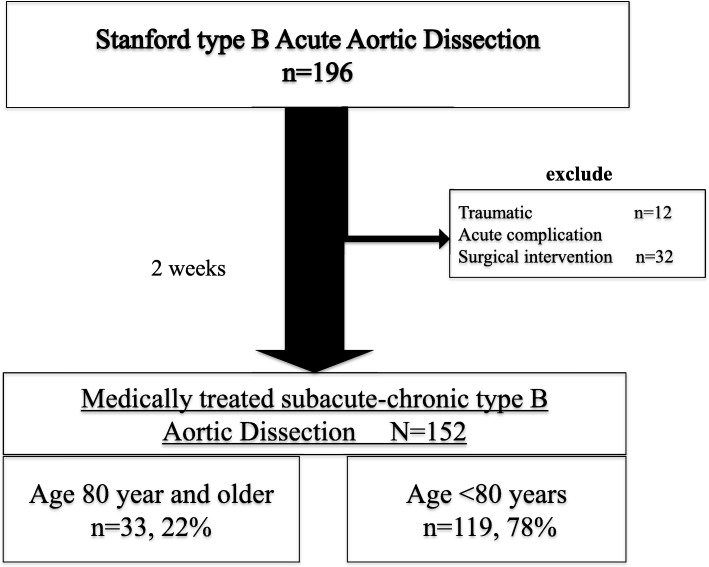
Summary flow diagram of patient disposition

Two centers in Yamagata prefecture participated in this study. Data were collected between July 2004 and October 2017 in Yamagata university hospital and between February 2016 and May 2018 in Nihonkai General hospital. A total of 196 patients were admitted to these two hospitals with a diagnosis of type B acute aortic dissection. In all patients, the time of onset of acute type B aortic dissection was clear. Patients were excluded if they developed acute complications in two weeks (32 patients) or if they had traumatic acute type B aortic dissection (12 patients). In the acute complication group, two patients (6.3%) were octogenarians. Both patients were treated with graft replacement (one for aortic rupture and the other for malperfusion). Both patients survived after surgical treatment and were discharged from the hospital. The remaining 152 patients treated medically for two weeks were enrolled in this study *(*Fig. 1*)*. After acute 2 weeks medical treatment, patients were divided into two groups: age 80 year and older group (Group O, *n* = 33, 22%) and a group < 80 years of age (Group U, *n* = 119, 78%). Only one patient was over 90-year-old, in Group O. Acute uncomplicated Stanford type B aortic dissection is defined as the absence of malperfusion or signs of (early) disease progression [2] and presenting within 14 days of symptom onset. We divided the time course of aortic dissection into acute (< 14 days), sub-acute (15–90 days), and chronic (> 90 days) phases. All patients were treated initially with medical management and followed up according to the standard clinical regimen. All patients received an arterial pressure catheter, central venous catheter, intravenous agents and continuous monitoring of vital signs. For blood pressure control, all patients received an intravenous calcium-channel blocker, nitroglycerine, β-blockers or a combination of these after admission. Systolic blood pressure was controlled to under 120 mmHg with careful observation of urine output from 2 weeks after onset. After 2 weeks, blood pressure was controlled to under 130 mmHg. On admission, patients were treated in the intensive care unit (ICU) or a high care unit (HCU). All patients underwent contrast computed tomography (CT) scanning at emergency admission, and on the 1st and 7th days after admission. All patients were administered oral medications starting on the 1st day after CT screening, and were encouraged to take a short walk starting the 7th day after onset. Patients were eligible for discharge 4 weeks after onset. That protocol was made by Japanese guidelines as a reference [6]. During follow-up, patients who had an aortic adverse event, despite medical management, underwent aortic interventions. The term “aortic adverse event” includes enlargement of the aortic diameter (≥ 55 mm enlargement and/or ≥ 5 mm enlargement in half a year), malperfusion, aortic rupture, and type A aortic dissection.

The status of the false lumen on imaging was classified as patent if flow was present in the absence of thrombus, as partially thrombosed if both flow and thrombus were present, or as completely thrombosed if no flow was present [7]. The judgement of the “thrombosed” or “partial thrombosed” were checked in the late phase of enhanced CT-scans.

Chronic obstructive pulmonary disease and connective tissue disorders were documented if they were present as diagnosis on the initial inpatient documentation. The aortic diameters during follow-up were compared with the maximum aortic diameter at baseline. For each patient, the slope of aortic growth was calculated individually based on the baseline maximum aortic diameter measurements and all repeated follow-up diameters (aortic growth rate).

### Follow-up

CT angiography images at presentation and all follow-up CT scans were reviewed for all patients. The standard scan regimen was as follows: at symptom onset, at discharge, 3 or 6 months after discharge, 12 months after discharge, and yearly thereafter. The scan regimen differed for individual patients, depending on findings. In all studies, radiologic signs of adverse events, such as organ malperfusion or rupture, as well as imaging evidence of pathology resolution, were reviewed. A total of 150 CT analysis were available for routine surveillance. Follow-up CT was absent in 2 patients under 80 years-old.

### Statistical methods

Baseline patient characteristics for the total study population and for Group O and Group U are described as absolute numbers and percentages for categorical variables and as mean (± standard deviation). Differences between groups were evaluated with the χ^2^ test for populations and the *t* test for independent samples of continuous variables (if normally distributed). Cumulative probabilities of event-free survival from aortic adverse events at 1 year, 5 years, and 10 years after onset were calculated with Kaplan-Meier survival analysis. The event-free survival curves of Groups O and U were compared with the log-rank test. Aortic-related death-free survival curves of Groups O and U were compared with the log-rank test. Analyses were conducted with JMP software, version 10 (SAS Institute).

## Results

### Demographics characteristics

Baseline patient characteristics are shown in Table 1. The characteristics of elderly patients (Group O) were smaller with a thinner body. We did a comparison using the aortic size index [8], but no statistically significant difference was obtained between the two groups. There were no other significant differences in the patients background between the both groups *(*Tables 1, 2*)*.

**Table 1:** Baseline patient characteristics

Characteristic	Total (*N* = 152)	≥80 years (*n* = 33)	< 80 years (*n* = 119)	*p* Value
Age, y, Mean ± SD	69.9 ± 10.9	84.8 ± 3.3	65.4 ± 10.1	< 0.0001
Male, %	71 (108 of 152)	61 (20 of 33)	74 (88 of 119)	0.192
Height, cm, Mean ± SD	162.0 ± 9.5	154.0 ± 7.9	164.2 ± 10.0	< 0.0001
Weight, kg, Mean ± SD	61.2 ± 13.3	49.4 ± 7.7	64.3 ± 14.4	< 0.0001
BMI (kg/m^2^), Mean ± SD	23.1 ± 4.2	20.9 ± 2.9	23.8 ± 4.5	0.0007
Marfan syndrome, %	2.6 (4 of 152)	0 (0 of 33)	3.4 (4 of 119)	0.5772
COPD, %	35 (53 of 150)	33 (11 of 33)	36 (42 of 117)	0.839
Follow up period, months, Mean ± SD	37 ± 43	37 ± 38	37 ± 44	0.995

*SD* Standard deviation

*BMI* Body mass index

*COPD* Chronic obstructive pulmonary disease

**Table 2:** Initial computed tomography findings

Characteristic	Total (*N* = 152)	≥80 years (*n* = 33)	< 80 years (*n* = 119)	*p* Value
Aortic diameter, mm				
On admission	38 ± 7	38 ± 7	38 ± 7	0.917
During follow-up (Max diameter)	40 ± 9	39 ± 8	40 ± 9	0.500
Aortic growth rate (mm/6 months)	3.0 ± 8.5	4.4 ± 9.6	2.7 ± 8.1	0.315
Aortic size index, cm/m^2^	3.9 ± 18.7	2.6 ± 0.5	4.3 ± 21.1	0.667
Number of intimal tears (patent false lumen)	2.4 ± 1.2	2.0 ± 1.2	2.4 ± 1.2	0.315
Thrombosed false lumen,%	59 (89 of 152)	67 (22 of 33)	56 (67 of 119)	0.323
Perfused with partial thrombosis, %	17 (26 of 152)	12(4 of 33)	19 (22 of 119)	0.601

### Aortic adverse events

During follow-up, a total of 9 patients (27%) in Group O and 44 patients (37%) in Group U experienced an aortic adverse event (*P* = 0.409). The 3-month, 2-year, and over 2 years occurrences of aortic-related events after onset were 5, 3, and 1, respectively, in Group O and 15 21, and 8 in Group U. In both groups, the most common aortic-related adverse event was aortic enlargement (7patients [21%] in Group O and 31 patients [27%] in Group U [*P* = 0.653] *(*Table 3*)*. Univariate analysis showed that Group O patients had a numerically higher growth rate (4.4 ± 9.6 mm/6 months) than Group U patients (2.7 ± 8.1 mm/6 months), but the difference was not statistically significant (*P* = 0.315). One patient died from an adverse aortic event in Group O; an 84-year-old man died of aorto-bronchial fistulation 2 months after onset. He had dementia and could not stand by himself, and his family did not request surgical intervention. In Group U, three patients (5%) died of aortic rupture, and two patients died after graft replacement.

**Table 3:** Outcomes of Aortic-Related Events and Mortality

Characteristic	Total (*N* = 152)	≥80 years (*n* = 33)	< 80 years (*n* = 119)	*p* Value
Aortic-related event, %	35 (53 of 152)	27 (9 of 33)	37 (44 of 119)	0.409
Rupture, %	2.6 (4 of 152)	3 .0(1 of 33)	2.5 (3 of 119)	0.872
Type A aortic dissection, %	1.3 (2 of 152)	3 (1 of 33)	0.8 (1 of 119)	0.388
Malperfusion, %	0.8 (1 of 152)	0 (0 of 33)	0.8 (1 of 119)	0.597
Aortic enlargement, %	25 (38 of 150)	21 (7 of 33)	27 (31 of 117)	0.653
Increase ≥5 mm/6 months, %	18 (27 of 150)	12 (4 of 33)	20 (23 of 117)	0.444
Aortic intervention, %	22 (33 of 152)	9 (3 of 33)	25 (30 of 119)	0.047
Aortic-related death, %	4 (6 of 152)	3 (1 of 33)	4 (5 of 119)	0.760

### Aortic intervention

In Group O, 3 patients (9%) underwent aortic interventions. Two patients were treated with graft replacement, and one patient was treated with endovascular aortic repair. In Group U, aortic interventions were needed in 30 patients (25%) including 11 patients with graft replacement, 16 with endovascular repairs, 2 patients with hybrid (open repair and endovascular) repair and 1 patient with an extra-anatomical bypass. The survival rate after surgical intervention was 90% (27 of 30) in Group U and 100% (3 of 3) in Group O. (No aortic adverse events recurred postoperatively).

### Long-term outcomes

**Fig. 2:**
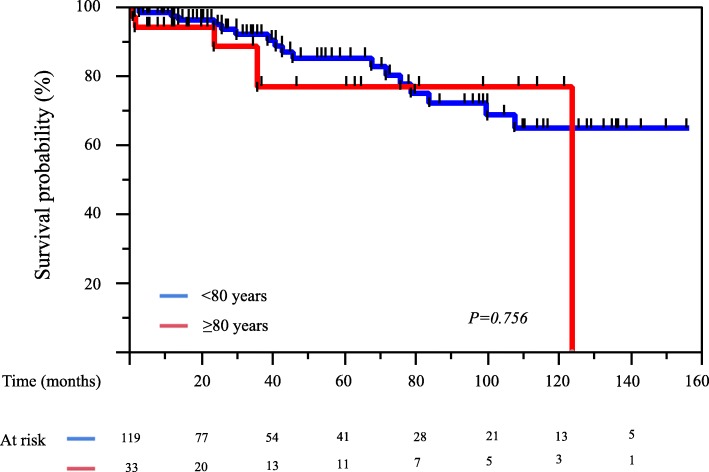
Kaplan-Meier curves for overall survival of 152 patients with uncomplicated chronic type B aortic dissection, compared with age 80 year and older patients and patients < 80 years of age

**Fig. 3:**
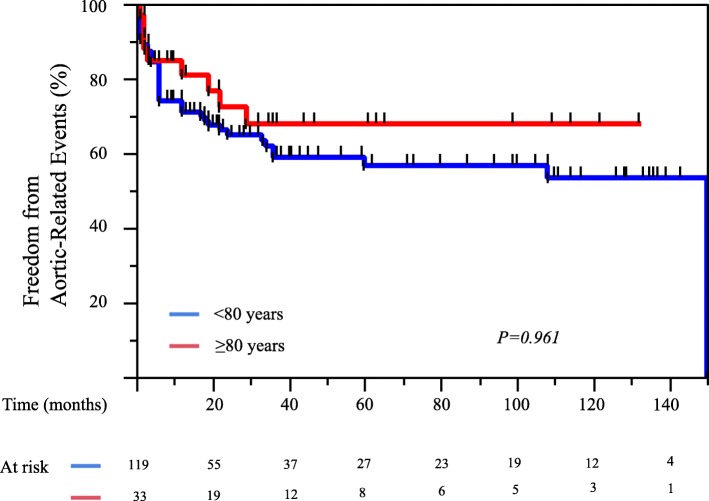
Kaplan-Meier curves for aortic-related event-free survival of 152 patients with uncomplicated chronic type B aortic dissection: age 80 year and older patients and patients < 80 years of age

**Fig. 4:**
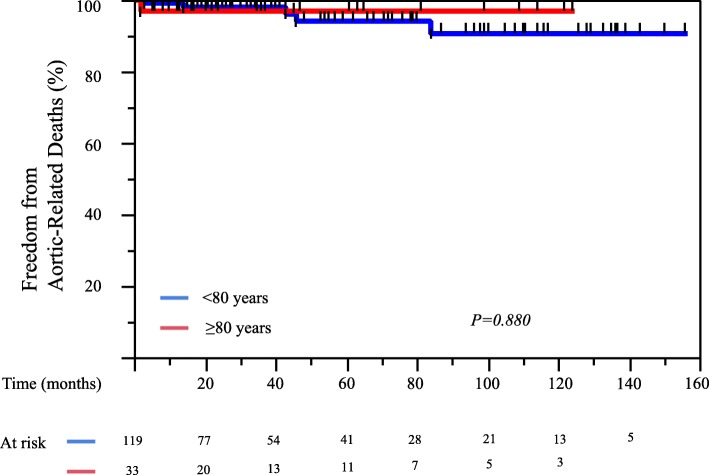
Kaplan-Meier curves for freedom from aortic-related death of 152 patients with uncomplicated chronic type B aortic dissection: age 80 year and older patients and patients < 80 years of age

The 1-, 5-, and 10-year overall survival rates for Group O patients were 94, 77, and 77%, respectively, compared with 97, 85, and 65%, respectively, in Group U (*P* = 0.756) (Fig. 2). The 1-, 5-, and 10-year aortic-related event-free rates for Group O were 81, 68, and 68%, respectively, compared with 71, 57, and 53%, respectively, in Group U (*P* = 0.961) (Fig. 3). The 1-, 5-, and 10-year aortic-related death-free survival rates for Group O were 97, 97, and 97%, respectively, compared with 99, 94, and 91%, respectively, in Group U (*P* = 0.880) (Fig. 4). In addition, 91% patients (30/33) in the Group O and 93% patients (111/119) in the Group U were followed up for at least 1 month.

The Group O patients had less surgical intervention (*P* = 0.047, Table 3), but aortic related death did not differ between the two groups.

## Discussion

The 2014 European Society of Cardiology guidelines [2] state that in uncomplicated type B aortic dissection, TEVAR should be considered to prevent aortic expansion (class IIa). Preemptive TEVAR has now gained broad approval as the treatment of choice in subacute uncomplicated type B aortic dissection. In the ADSORB trial [9], a randomized trial on acute dissection that compared best medical treatment (BMT) with BMT plus thoracic stent grafting of the primary entry tear in patients with acute uncomplicated type B dissection, good short-term results were demonstrated. The International Registry of Acute Aortic Dissection has reported good results for TEVAR for type B acute aortic dissection [10]. Preemptive TEVAR of the acute and subacute phases of type B aortic dissection may prevent late complications and provide favorable late outcomes. However, complications during the acute phase were more common in patients who underwent TEVAR compared with patients given medication alone [10]. Another potentially important risk factor applicable to the use of TEVAR for dissection is the occurrence of stent graft-induced new entry [11], retrograde type A aortic dissection [12], and thoracoabdominal aneurysmal dilatation [13]. As mentioned above, many problems remain, such that some cases require reintervention. Especially in elderly patients, a higher intervention risk is present due to their frailty and worse aortic properties compared with younger patients. Although preemptive TEVAR in octogenarians may appear to be prudent because short and long-term outcomes of medical treatment for type B aortic dissection showed good mortality and morbidity, in our study, patients in Group O experienced less surgical intervention (Group O vs Group U = 9% vs 25%, *P* = 0.047), but the occurrence of late aortic-related deaths was similar in both groups. Thus, preemptive TEVAR should not be applied to undifferentiated younger and elderly patients with uncomplicated type B aortic dissection.

Recently, various factors predicting long-term adverse aortic events and mortality has been reported in patients with type B aortic dissection at follow-up; dissection entry tears >10mm [14, 15], the number of vessels originating from the false lumen [16] and the number of intercostal arteries [15]. The younger age [5, 7, 17] was known as the one of the major predictive factor of aortic adverse events. However, no study has revealed the mechanism underlying slower aortic growth and rupture in elderly patients. In this study, octogenarians had a higher incidence of thrombosed false lumen (67% vs 56%) and a smaller number of intimal tears (2.0 ± 1.2 vs 2.4 ± 1.2, *P* = 0.315). Although there was no statistical difference between 2 groups, some kind of physiological properties of octogenarians might be related to these findings.

In INSTEAD-XL [1], *Nienanber* found that preemptive TEVAR appeared to be useful for younger patients; for some very old patients, the benefit may not emerge during their expected lifetime, and severe comorbidities may still favor medical management. In elderly patients, an etiological difference in type B aortic dissection may be present compared with younger patients. We suggest that preemptive TEVAR is not required as a uniform application, and that details of each patient’s background should be considered.

### Limitations

Our study is limited by its retrospective design, its relatively small number of patients, incomplete follow-up (7%), and varying number of CT scans among patients.

## Conclusions

Through the observantion of the natural history of type B aortic dissection, Patients aged 80 years and older who had medical treatment for acute and subacute type B dissection had excellent outcomes in chronic phase. The elderly patients had less surgical intervention, but aortic related death did not differ from younger patients.

## Abbreviations

BMI: Body Mass Index
BMT: best medical treatment
COPD: Chronic Obstructive Pulmonary Disease
CT: computed tomography
HCU: High Care Unit
ICU: Care Unit
SD: Standard Deviation
TEVAR: thoracic endovascular aortic repair

## References

[1] Christoph AN, Stephan K, Herve R, Holger E, Tim CR, Guenther K (2013). Endovascular repair of type B aortic dissection long-term results of the randomized investigation of stent grafts in aortic dissection trial. Circ Cardiovasc Interv.

[2] Raimund E, Victor A, Catherine B, Eduardo B, RobertoDB, Holger E, et al. 2014 ESC guidelines on the diagnosis and treatment of aortic disease. Eur Heart J 2014;35,2873–2926.

[3] Akutsu K, Nejima J, Kikuchi K, Sasaki K, Ochi M, Tanaka K (2004). Effects of patent false lumen on long-term outcome of type B acute aortic dissection. Eur J Cardiothorac Surg.

[4] Jip LT, Jasper WK, Frederik HWJ, Joost AH, Hence JV, Frans LM (2013). Morphologic predictors of aortic dilatation in type B aortic dissection. J Vasc Surg.

[5] Luebke T, Brunkwall J (2014). Type B aortic dissection: a review of prognostic factors and meta-analysis of treatment options. Aorta.

[6] Komoto S, Ishimaru A, Ueda Y, Ohki T, Okita Y, Ogino H, et al. Guidelines for Diagnosis and Treatment of Aortic Aneurysm and Aortic Dissection. 2011.http://www.j-circ.or.jp/guideline/pdf/JCS2011_ takamoto_h.pdf(cited 2013 July 30).

[7] Tsai TT, Evangelista A, Nienaber CA, Myrmel T, Meinhardt G, Cooper JV (2007). Partial thrombosis of the false lumen in patients with acute type B aortic dissection. N Engl J Med.

[8] Davies RR, Gallo A, Coady MA, Tellides G, Botta DM, Burke B (2006). Novel measurement of relative aortic size predicts rupture of thoracic aortic aneurysms. Ann Thorac Surg.

[9] Hughes GC (2015). Management of acute type B aortic dissection; ADSORB trial. J Thorac Cardiovasc Surg.

[10] Fattori R, Montgomery D, Lovato L, Kische S, Di Eusanio M, Ince H (2013). Survival after endovascular therapy in patients with type B aortic dissection: a report from the international registry of acute aortic dissection (IRAD). JACC Cardiovasc Interv.

[11] Zhihui D, Weiguo F, Yuqi W, Chunsheng W, Zhiping Y, Daquiao G, et al. Stent graft-induced new entry after endovascular repair for Stanford type B aortic dissection. J Vasc Surg 2010;52:1450–1458. 10.1016/j.jvs.2010.05.121 20800417

[12] Hunter MR, Christopher AD, Daniel O, Kristofer M, Charlton O, Anthony LE (2016). Predictors of intervention and mortality in patients with uncomplicated acute type B aortic dissection. J Vasc Surg.

[13] Richard PC, Mark FC (2016). Thoracic endovascular aneurysm repair for uncomplicated type B dissection. J Vasc Surg.

[14] Cambria RP, Conrad MF (2016). Thoracic endovascular aneurysm repair for uncomplicated type B dissection. J Vasc Surg.

[15] Schwartz SI, Durham C, Clouse WD, Patel VI, Lancaster RT, Cambria RP (2018). Predictors of late aortic intervention in patients with medically treated type B aortic dissection. J Vasc Surg.

[16] Kamman AV, Brunkwall J, Verhoeven EL, Heijmen RH, Trimarchi S (2017). Predictors of aortic growth in uncomplicated type B aortic dissection from the acute dissection stent grafting or best medical treatment (ADSORB) database. J Vasc Surg.

[17] van Bogerijen GH, Tolenaar JL, Rampoldi V, Moll FL, van Herwaarden JA, Jonker FH (2014). Predictors of aortic growth in uncomplicated type B aortic dissection. J Vasc Surg.

